# Novel NKG2D-directed bispecific antibodies enhance antibody-mediated killing of malignant B cells by NK cells and T cells

**DOI:** 10.3389/fimmu.2023.1227572

**Published:** 2023-10-27

**Authors:** Sebastian Lutz, Katja Klausz, Anca-Maria Albici, Lea Ebinger, Lea Sellmer, Hannah Teipel, André Frenzel, Anna Langner, Dorothee Winterberg, Steffen Krohn, Michael Hust, Thomas Schirrmann, Stefan Dübel, Regina Scherließ, Andreas Humpe, Martin Gramatzki, Christian Kellner, Matthias Peipp

**Affiliations:** ^1^Department of Transfusion Medicine, Cell Therapeutics and Hemostaseology, University Hospital, Ludwig Maximilians University (LMU) Munich, Munich, Germany; ^2^Division of Antibody-Based Immunotherapy, Department of Medicine II, Kiel University, Kiel, Germany; ^3^YUMAB GmbH, Braunschweig, Germany; ^4^Technische Universität Braunschweig, Institut für Biochemie, Biotechnologie und Bioinformatik, Abteilung Biotechnologie, Braunschweig, Germany; ^5^Department of Pharmaceutics and Biopharmaceutics, Kiel University, Kiel, Germany

**Keywords:** bispecific antibody, phage display, antibody therapy, NKG2D, FcγRIIIA, CD20, lymphoma

## Abstract

The activating receptor natural killer group 2, member D (NKG2D) represents an attractive target for immunotherapy as it exerts a crucial role in cancer immunosurveillance by regulating the activity of cytotoxic lymphocytes. In this study, a panel of novel NKG2D-specific single-chain fragments variable (scFv) were isolated from naïve human antibody gene libraries and fused to the fragment antigen binding (Fab) of rituximab to obtain [CD20×NKG2D] bibodies with the aim to recruit cytotoxic lymphocytes to lymphoma cells. All bispecific antibodies bound both antigens simultaneously. Two bibody constructs, [CD20×NKG2D#3] and [CD20×NKG2D#32], efficiently activated natural killer (NK) cells in co-cultures with CD20+ lymphoma cells. Both bibodies triggered NK cell-mediated lysis of lymphoma cells and especially enhanced antibody-dependent cell-mediated cytotoxicity (ADCC) by CD38 or CD19 specific monoclonal antibodies suggesting a synergistic effect between NKG2D and FcγRIIIA signaling pathways in NK cell activation. The [CD20×NKG2D] bibodies were not effective in redirecting CD8+ T cells as single agents, but enhanced cytotoxicity when combined with a bispecific [CD19×CD3] T cell engager, indicating that NKG2D signaling also supports CD3-mediated T cell activation. In conclusion, engagement of NKG2D with bispecific antibodies is attractive to directly activate cytotoxic lymphocytes or to support their activation by monoclonal antibodies or bispecific T cell engagers. As a perspective, co-targeting of two tumor antigens may allow fine-tuning of antibody cancer therapies. Our proposed combinatorial approach is potentially applicable for many existing immunotherapies but further testing in different preclinical models is necessary to explore the full potential.

## 1 Introduction

Since approval of rituximab in 1997 antibody-based immunotherapy has become the fourth pillar in the treatment of cancer besides surgery, radiation and chemotherapy. However, despite this success story not all patients benefit and relapse is still a serious issue (1). Therefore, further development and optimization of antibody therapy is a major objective in current translational research. Recruitment of effector cells plays a crucial role for the efficacy of therapeutic antibodies as revealed in murine tumor models and by observations in patients (2–5). Thus, approaches improving this effector function are very attractive.

This mechanism of action is based on the interaction of the antibody fragment crystallizable (Fc) domain and activating Fcγ receptors expressed on effector cells, resulting in antibody-dependent cellular phagocytosis (ADCP) or antibody-dependent cell-mediated cytotoxicity (ADCC). Especially in the treatment of minimal residual disease antibody therapy is promising, since at low tumor burden high effector-to-target cell ratios (E:T ratios) can be expected. However, recruitment of effector cells through therapeutic antibodies is often insufficient in patients (6). To overcome this limitation various strategies have been developed to improve effector cell engagement, for example by optimizing the Fc-domain of immunoglobulin G (IgG) antibodies by Fc-engineering (7), combining monoclonal antibodies (mAbs) with immune stimulatory molecules (8), or using bispecific antibodies (bsAbs) (9).

BsAbs for effector cell recruitment represent a promising class of therapeutic agents in cancer immunotherapy. These molecules combine at least two antigen binding moieties of different specificity, the first to target an antigen on tumor cells and the second to trigger an activating receptor on immune cells. Especially natural killer (NK) cells and T cells can be activated by these agents efficiently, when targeting e.g. Fcγ receptor (FcγR) IIIA (CD16a), CD3 or the γδ T cell receptor (TCR), respectively (9–11). Besides chimeric antigen receptor (CAR) T cells, CD3 bsAbs constitute the most powerful agents for induction of major histocompatibility complex (MHC)-independent T cell responses against cancer (12). In 2015, the bispecific T cell engager (BiTE) blinatumomab, a [CD19×CD3] bsAb received marketing approval in both the US and the EU in the treatment of relapsed or refractory B cell precursor acute lymphoblastic leukemia (13, 14). More recently, with teclistamab and mosunetuzumab-axgb two additional T cell engagers received approval in the treatment of multiple myeloma and B cell lymphoma, respectively (15, 16). Yet, NK and T cells express various receptors with stimulatory or co-activating functions, which also have great potential to act as trigger molecules for bsAbs.

One candidate in this aspect is the activating receptor natural killer group 2 member D (NKG2D; CD314), a C-type lectin-like receptor, that plays a key role in immunosurveillance of tumors and pathogens (17, 18). In humans, NKG2D is expressed by NK cells and T cells and recognizes “induced-self proteins”, which are frequently expressed at the cell surface after viral infection or malignant transformation (19, 20). Human NKG2D ligands include major histocompatibility complex (MHC) class I-related chain (MIC) A and B as well as UL16-binding proteins (ULBP) 1-6. Recognition of these danger signaling antigens results in cell activation through an intracellular signaling pathway via the NKG2D-associated adapter protein DNAX-activating protein of 10 kDa (DAP10) (21). In NK cells, this signal leads to induction of natural cytotoxicity without further co-stimulation (22). In addition to NK cells, in humans NKG2D is also expressed on CD8^+^ αβ T cells, γδ T cells, NKT cells as well as subsets of CD4^+^ T cells and mediates stimulating or co-activating signals. Previous studies have shown that co-stimulation by NKG2D regulates priming, proliferation and function of cytotoxic T cells (23, 24). After prolonged stimulation with IL-2 or IL-15, T cells were also able to kill target cells TCR-independent via NKG2D activation (25, 26). Diefenbach and colleagues showed that the expression of NKG2D ligands triggered adaptive immune responses, which were dependent on activation of NK cells and T cells (27).

During cancer progression many tumors escape this immunosurveillance mechanism through downregulation or proteolytic shedding of NKG2D ligands (19, 28, 29). Recently, leukemic stem cells have been described to lack NKG2D ligand expression, thereby evading destruction by NK cells (30). Therefore, different strategies were pursued to restore NKG2D-mediated recognition of malignant cells. In a recent study, anti-MICA and anti-MICB antibodies were used to inhibit shedding of these ligands, resulting in enhanced NK cell cytotoxicity through NKG2D and additional FcγRIIIA activation (31). As NKG2D is expressed on NK cells as well as on T cell subsets, it may also represent a promising target for antibody-based immunotherapy. Fusion proteins of antibody fragments and NKG2D ligands were employed to coat tumor cells with the danger signal. Thus, a bispecific immunoligand, which consists of the natural NKG2D-ligand ULBP2 fused to a scFv targeting CD138 expressed on multiple myeloma (MM) cells, showed promising results *in vitro* and in a xenograft mouse model (32). With a similar construct targeting CD20, we have shown that bispecific immunoligands engaging NKGD2 trigger NK cell cytotoxicity and synergistically enhance NK cell-mediated ADCC by therapeutic antibodies (33, 34). In addition, ULBP2 containing immunoligands were demonstrated to promote CD8^+^ T cell activation when combined with a bispecific CD3 T cell engager *in vitro* (35). Similar approaches have been described using MICA-based fusion proteins (36). Furthermore, a bsAb with specificities for NKG2D and SLAM family member 7 (SLAMF7; CS-1; CD319) was shown to exert therapeutic effects in preclinical models of MM (37). In recent studies, bispecific antibodies targeting Her2 and NKG2D were generated using scFv or VHH antibodies and were shown to trigger efficient target cell killing (38, 39). Interestingly, such bispecific molecules were also able to redirect genetically modified T or NK cells engineered to express a NKG2D-based chimeric antigen receptor against Her2 expressing cancer cells (39).

In this study, novel human NKG2D antibodies were isolated by phage display (40) and used to develop costimulatory bsAbs targeting CD20^+^ lymphoma cells. The use of antibodies as compared to natural ligands as triggering device may circumvent potential production issues related to a complex glycosylation profile present on natural ligands. The molecules were biochemically characterized and screened for their potential to activate effector cells. Promising candidates were utilized to further improve the cytotoxic potency of NK cells in combination with tumor specific monoclonal antibodies as well as a bispecific T cell engager, respectively. This strategy could improve existing clinically used antibody therapies by enhancing the cytotoxicity of both NK and T effector cell populations in a co-stimulatory approach.

## 2 Materials and methods

### 2.1 Phage display

Phage display experiments were performed as described previously (41). Naïve antibody gene libraries HAL7 and HAL7b were used for bio-panning against a recombinant human NKG2D-Fc fusion protein. NKp30-Fc was employed as a control for negative selection and competition (34). A total of 276 clones from HAL7 and 230 clones from HAL7b were repackaged with M13K07 helper phage and then screened by a monoclonal phage ELISA (42). Briefly, a total of 50 ng/well NKG2D-Fc and NKp30-Fc antigens were coated in 96-well MTPs (High Binding, Costar) in PBS over night at 4°C. Subsequent blocking was performed with 2% (w/v) skim milk powder in PBS with 0.05% Tween-20 (M-PBST) for 1 h at room temperature. All following washing steps were performed three times with PBST using an ELISA washer. A total of 100 µL/well of diluted antibody-phage carrying different scFv clones from the panning against NKG2D-Fc were prepared in M-PBST and incubated on the antigen-coated wells. Detection was performed with a mouse anti-M13 antibody HRP conjugate (GE Healthcare) followed by goat anti-mouse Fc specific secondary antibody horseradish peroxidase (HRP) conjugate (Sigma). Finally, substrate TMB (3,3′,5,5′-tetramethylbenzidine) was added, and the color reaction was stopped by adding 100 µL 1 N sulfuric acid. Absorbances were measured at 450 nm (with 620 nm reference wavelength) using an ELISA reader (SUNRISE, Tecan).

### 2.2 Sequencing, sequence analysis

Sanger sequencing was used to identify different NKG2D-specific scFvs and to verify DNA sequences. Sequences were analyzed using VBASE2 (43).

### 2.3 Cell culture

Raji cells (DSMZ) were maintained in RPMI 1640 Glutamax-I medium (Thermo Fisher Scientific) supplemented with 10% fetal calf serum (FCS; Thermo Fisher Scientific), 100 U/mL penicillin and 100 mg/mL streptomycin (Thermo Fisher Scientific). GRANTA-519 (DSMZ) and Lenti-X 293T cells (Takara Bio Europe/Clontech) were cultured in Dulbecco’s modified Eagle medium-Glutamax-I medium (Thermo Fisher Scientific) supplemented with 10% FCS, 100 U/mL penicillin and 100 µg/mL streptomycin. Chinese hamster ovary (CHO)-S, suspension-adapted CHO cells (Thermo Fisher Scientific) were kept in CD CHO-Medium (Thermo Fisher Scientific) containing 1% GlutaMax-I (200 mM L-Ala-L-Gln, Gibco/Thermo Fisher Scientific) and 1% HT Supplement for maintenance and in CD OptiCHO (Thermo Fisher Scientific) supplemented with 1% Pluronic-F68, 1% GlutaMax-I and 1% HAT-Supplement (CHO production medium) for antibody production.

### 2.4 Cloning, expression and purification of bsAbs and antibody derivatives

For construction of the heavy chain derivatives of bibodies, DNA sequences for the different anti-NKG2D scFvs were ligated as NcoI/NotI cassettes into expression vector pΔIRES-RTX-VH-CH1 (unpublished). This is a derivative of vector pIRES-ZSK Green, in which both the internal ribosomal entry site and the GFP coding sequence had been replaced by sequences coding for the rituximab VH leader, rituximab VH chain, the IgG1 CH1 domain and the antibody`s upper hinge region. For production of small quantities for [CD20×NKG2D] bibody screening, Lenti-X 293T cells were transiently co-transfected with expression vectors encoding either the bibodies’ heavy chain derivative or the rituximab light chain (44) by the calcium phosphate method as described earlier (10). Selected clones were also expressed transiently in CHO-S cells by electroporation using MaxCyte STX electroporation system (MaxCyte) (45). Transfected cells were cultured in CD OPTICHO medium containing 1% pluronic-F68 (Thermo Fisher Scientific), 1% Glutamax-I (Thermo Fischer Scientific) and 1% HT supplement (Thermo Fisher Scientific) at 32°C, 5% CO_2_ and 143 rpm. After 24 h, sodium butyrate (Sigma) was added to a final concentration of 15 µM and 3.5% (v/v) feed stock solution, which contained 70% CHO CD Efficient Feed A Stock Solution (Thermo Fisher Scientific), 14% Yeastolate TC UF (Becton Dickinson), 3.5% GlutaMax-I(200 mM) and 12.5% Glucose (450 g/L, Sigma), was supplemented daily. The production was terminated when cell viability decreased below 50% and the cell culture supernatant was collected. Bibodies were purified by affinity chromatography with CaptureSelect IgG-CH1 affinity matrix (Thermo Fisher Scientific) following manufacturer’s instructions. BiTE-like constructs [CD19×CD3] and [HER2×CD3], which both are based on the CD3 scFv moiety from blinatumomab (WO2005/040220), were expressed and purified as described previously (11). Fusion proteins NKG2D-Fc, NKp30-Fc were produced as previously published (33). After extensive dialysis against phosphate-buffered saline (PBS, Invitrogen) the molecules were stored at 4°C until usage. For selected experiments multimers were removed by size exclusion chromatography (**Supplementary Figure 1**).

### 2.5 Sodium dodecyl sulfate polyacrylamide gel electrophoresis

Separation and detection of recombinant bsAbs were performed by SDS-PAGE under reducing or non-reducing conditions, according to standard procedures. Proteins were analyzed by Coomassie staining (Coomassie brilliant blue G250 solution, Carl Roth GmbH). The concentration of purified bsAbs was estimated against a standard curve of rituximab (Roche).

### 2.6 Size exclusion chromatography

Size exclusion chromatography was performed on an ÄKTA purifier (GE Healthcare) using PBS as running buffer at a constant flow rate of 1 ml/min. Thyroglobulin (669 kDa, Cytiva), aldolase (158 kDa; Cytiva) and ribonuclease A (13.7 kDa; Cytiva) were used for calibration.

### 2.7 Flow cytometry

Flow cytometry experiments were performed on a Navios flow cytometer (Beckman Coulter). Three hundred thousand cells were washed in PBS supplemented with 1% bovine serum albumin (Sigma-Aldrich) and 0.1% sodium-azide. Simultaneous binding was demonstrated by incubating Raji cells with the [CD20×NKG2D] bibodies (50 µg/mL), followed by a second incubation step with either NKG2D-Fc (100 µg/mL) or the control protein NKp30-Fc on ice for 60 minutes. Finally, the surface-bound complex was visualized by staining with polyclonal FITC-coupled anti-human IgG-Fc F(ab′)_2_ fragments (Beckman Coulter). Isolated NK or T cells were characterized by flow cytometry using FITC- or Pacific Blue-conjugated CD3 or CD8, APC-coupled CD56, PE-conjugated CD16 antibodies (Beckman Coulter) and corresponding isotype controls according to the manufacturer’s recommendations. CD19, CD20 and CD38 expression on target cells was analyzed analogously using PE or FITC-conjugated antibodies (Beckman Coulter).

### 2.8 Preparation of mononuclear cells and isolation of NK and T cells

All experiments were authorized by the Ethics Committee of the Kiel University (Kiel, Germany). Blood from donors was drawn after having received written informed consent. Preparation of MNC from peripheral blood of patients and healthy volunteers or from leukocyte reduction system chambers was performed via Ficoll-Paque PLUS density gradient (GE Healthcare). After centrifugation, MNC were collected at the Serum/Ficoll interface and remaining erythrocytes were removed by hypotonic lysis. NK cells and CD8^+^ αβ T cells were isolated from MNC by MACS technology via negative selection using NK cell isolation kit and CD8^+^ T cell isolation kit (Miltenyi), respectively, following the manufacturer’s protocols. In CD8^+^ T cell preparations any potentially remaining NK cells were removed in a secondary depletion step using CD56 MicroBeads (Miltenyi). Purified MNC were directly employed in functional assays. NK cells were cultured overnight at a density of 2×10^6^ cells/mL in RPMI 1640 Glutamax-I medium supplemented with 10% FCS, 100 U/mL penicillin and 100 mg/mL streptomycin. CD8^+^ T cells were kept at a density of 1×10^6^ cells/mL in RPMI 1640 Glutamax-I medium supplemented with 10% FCS, 100 U/mL penicillin and 100 mg/mL streptomycin and stimulated with IL-2 (300 U/mL) for 48 h prior to functional analysis.

### 2.9 Analysis of NK cell activation

One hundred thousand NK cells were co-incubated with equal numbers of GRANTA-519 cells in microtiter plates in a volume of 200 µL. The [CD20×NKG2D] bibodies at the indicated concentrations or PBS were added. After 4 h cells were stained with antibodies against CD69 (PE-conjugated, Beckman Coulter), CD56 (APC, Beckman Coulter), CD19 (FITC, Beckman Coulter) and CD3 (Pacific Blue, Beckman Coulter) and analyzed by flow cytometry. CD56-positive, CD3- and CD19-negative NK cells were gated and the expression levels of CD69 were determined.

### 2.10 Analysis of NK cell and T cell cytotoxicity

Cytotoxicity was analyzed in standard 4 h ^51^Cr release experiments, which were performed in 96-well microtiter plates in a total volume of 200 µL as described previously (10). Human NK cells, CD8^+^ T cells or MNC were used as effector populations at the indicated E:T ratios. The bispecific [CD20×NKG2D] bibodies, the antibodies daratumumab (Janssen), CD19-DE or trastuzumab (Roche) as a non-binding IgG1 control and the BiTE molecules [CD19×CD3] or [HER2×CD3] were analyzed at the indicated concentrations.

### 2.11 Statistical analysis and data processing

P-values were determined using repeated measures ANOVA and the Bonferroni post-test. The null hypothesis was rejected for p < 0.05. Statistical and graphical analyses were performed with GraphPad Prism 5.0 software. Synergy was analyzed by interpolating required antibody doses at distinct effect levels using GraphPad Prism 5.0 software and calculating combination index (CI) values using the formula CI_x_ = D_A_/D_xA_ + D_B_/D_xB_ (D_xA_ and D_xB_, dose of drugs A and B alone producing x% effect; D_A_ and D_B_, doses of drugs A and B in combination producing equal effects) (46). Synergistic effects were classified into strong synergy (CI = 0.1 – 0.3), synergy (CI = 0.3 – 0.7), moderate synergy (CI 0.7 – 0.85), slight synergy (CI = 0.85 – 0.95), additivity (CI = 1) and antagonism (CI > 1).

## 3 Results

### 3.1 Isolation of human NKG2D antibodies by phage display

**Figure 1:**
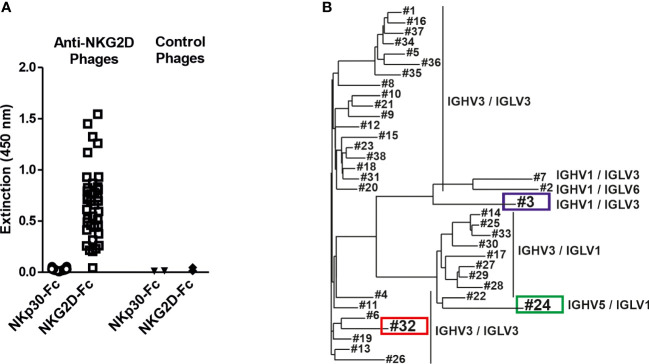
Isolation of NKG2D-specific human scFv antibodies and sequence analysis of the V regions. **(A)** ScFv phage, which had been isolated from a naïve antibody library by panning against the human NKG2D antigen, were analyzed for specific antigen binding by phage ELISA using an NKG2D-Fc fusion protein and the analogously constructed control protein NKp30-Fc. ScFv phage binding an irrelevant antigen were used as controls. **(B)** The isolated NKG2D-specific scFvs were grouped via sequence analysis into 3 different groups according to different germline gene segment families as well as their VH/VL combinations (detailed sequence information is available (47):). The further characterized clones #3 (blue) and #32 (red) and the later used control scFv #24 (green) are highlighted.

To generate novel human NKG2D-specific antibodies the two naïve human scFv antibody libraries HAL7 and HAL7b (41) were screened by bio-panning against a recombinant fusion protein consisting of the extracellular domain of human NKG2D and the human IgG1-Fc part. Binding analyses of isolated clones revealed that 38 different phage clones bound NKG2D-Fc, but not a control molecule consisting of the extracellular domain of natural killer protein 30 (NKp30-Fc, **Figure 1A**). Sequence analysis by aligning the variable (V) regions of the different antibodies revealed that the clones clustered in distinct groups according to primary sequence diversity. In addition, the individual clones could be assigned to different families of germline V gene segments, had various combinations of different variable heavy (V_H_) and variable light (V_L_) chains and could be divided into three groups (**Figure 1B**). The majority of all clones contained IGHV3, which has been reported to display the highest thermodynamic stability and yield of soluble protein (48). The largest group had combinations of IGHV3/IGLV3 (25 clones) which interestingly separated in two groups, followed by IGHV3/IGLV1 (9 clones). Furthermore, rare combinations of IGHV1/IGLV3 (2 clones), IGHV1/IGLV6 and IGHV5/IGLV1 (1 clone each) were identified.

### 3.2 Generation of bispecific antibodies

**Figure 2:**
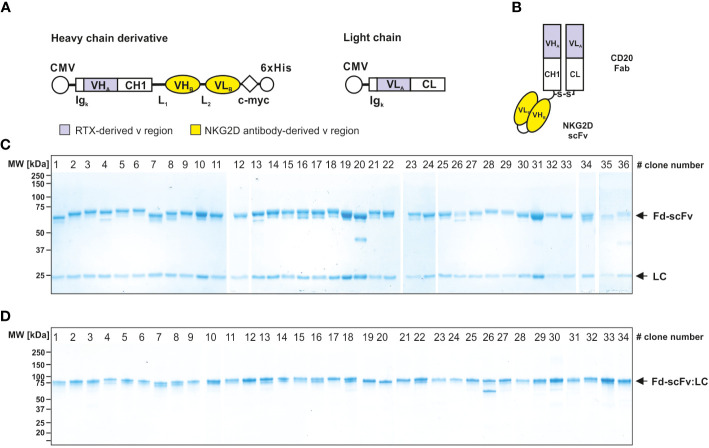
Generation and characterization of bispecific [CD20×NKG2D] bibodies. **(A)** Schematic illustrations of the expression cassettes of bispecific [CD20×NKG2D] antibodies in the bibody format (Fab-scFv). CMV, cytomegalovirus promotor; Ig_κ_, human Ig kappa secretion leader; VH_A_, VL_A_, sequences coding for the variable regions of the immunoglobulin heavy and light chains of the CD20 antibody rituximab (RTX), respectively; CH1, CL, sequences coding for the human immunoglobulin heavy chain constant region 1 and the human immunoglobulin kappa-light chain constant region, respectively; VH_B_, VL_B_, cDNA sequence coding for the variable heavy and light chain regions of the NKG2D-specific scFv; L_1_, L_2_, sequence coding for a linker peptides; c-myc, 6×His, sequence coding for the c-myc epitope and a hexahistidine tag, respectively. **(B)** Block structure of the produced bispecific antibodies in the bibody format. The NKG2D-specific scFvs were fused to a CD20 directed Fab. S-S, disulfide bridge. Purity and integrity of purified bispecific antibodies, consisting of a light chain (LC, approx. 25 kDa) and a heavy chain derivate (Fd-scFv, approx. 60 kDa), were analyzed by Coomassie stained SDS-PAGE under reducing (10% PAA) **(C)** and non-reducing conditions (4 – 15% PAA) **(D)**. Of note: Only 36 of the initially sequenced 38 NKG2D scFvs could be successfully expressed as recombinant protein. Bibodies 35 and 36 expressed at very low levels and were not analyzed in all assay conditions. The numbering of the lanes represents the clone numbers #1 - #36 introduced in **Figure 1**. One representative experiment out of three is shown.

The 38 isolated scFvs were processed into bispecific [CD20×NKG2D] antibodies. To ensure an efficient screening process we used the heterodimeric bibody format (49), which in this case consists of the fragment antigen binding (Fab) derived from the CD20 specific mAb rituximab, genetically fused to the different anti-NKG2D scFvs via a flexible glycine-serine-linker (**Figures 2A, B**). The resulting bibodies were transiently expressed and purified from cell culture supernatants via affinity chromatography. Thirty-six of the 38 individual anti-NKG2D scFvs were successfully produced in the bibody format. Two constructs did not show any expression and were not feasible for unknown reasons. Integrity and purity of the proteins were analyzed by Coomassie-stained SDS-PAGE under reducing and non-reducing conditions (**Figures 2C, D**, respectively).

### 3.3 Antigen binding and activation of NK cells

**Figure 3:**
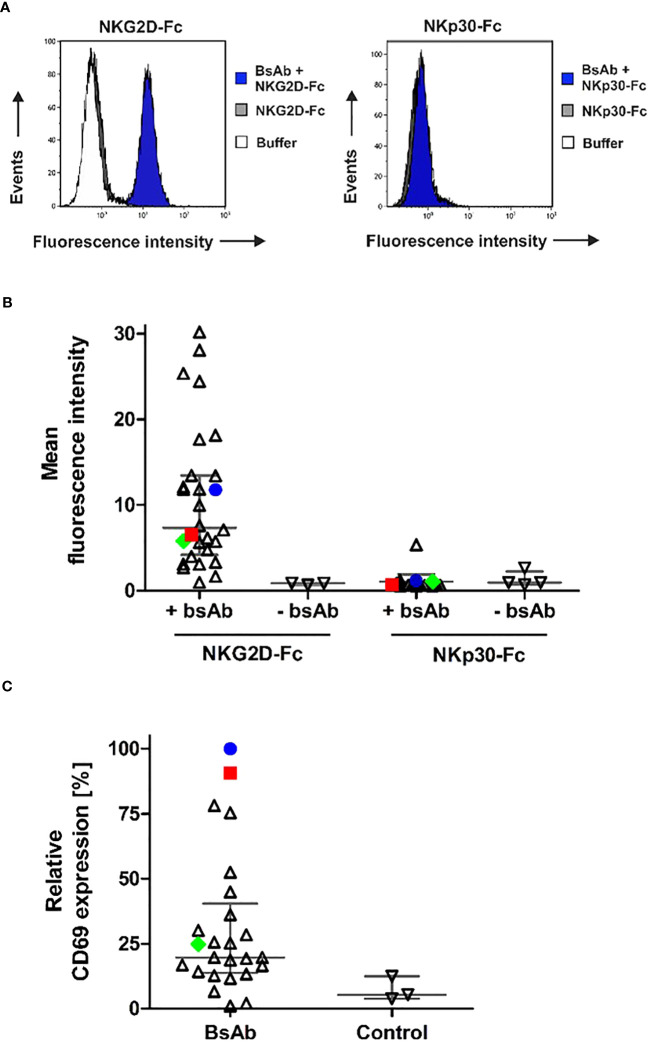
Simultaneous antigen binding of [CD20×NKG2D] bibodies and induction of NK cell activation. **(A)** CD20^+^ Raji lymphoma cells were first incubated with the different [CD20×NKG2D] bibodies and then reacted with NKG2D-Fc or with the control protein NKp30-Fc. Dual antigen binding of the bibodies was visualized by a FITC-coupled antibody against human Fc via flow cytometry. As a control, cells were incubated with either NKG2D-Fc or NKp30-Fc (control) in absence of the [CD20×NKG2D] bibodies or with the FITC-coupled detection antibody alone (buffer). The exemplary results are shown for the bibody [CD20×NKG2D#3], which specifically interacts with CD20 and NKG2D-Fc but not with NKp30-Fc. Note: in the left panel buffer control and NKG2D-Fc stainings are superimposed. **(B)** Abilities of various individual [CD20×NKG2D] bibody constructs containing different NKG2D scFv clones to simultaneously bind CD20 and NKG2D. Each data point represents an individual construct and indicates the mean fluorescence intensity value from three independent experiments. Horizontal lines show medians with interquartile range. The further characterized clones #3 (blue) and #32 (red) and the later used control scFv #24 (green) are highlighted. **(C)** NK cells were incubated with the [CD20×NKG2D] bibodies (10 µg/ml) in the presence of GRANTA-519 mantle cell lymphoma cells. As a control, NK cells and lymphoma cells were incubated in absence of a bibody. After 4 h, the induced expression of the activation marker CD69 was analyzed on CD56^+^/CD3^-^ NK cells via flow cytometry and mean fluorescent intensities were determined. Data points were normalized to CD69 expression induced by the bibody [CD20×NKG2D#3] and indicate mean values from 3 independent experiments. Horizontal lines indicate medians with interquartile range. The further characterized bibody constructs based on clones #3 (blue) and #32 (red) and the later used control construct #24 (green) are highlighted.

The binding abilities of the different [CD20×NKG2D] bibodies were analyzed by flow cytometry. In particular, the capacity of simultaneous binding to both antigens was determined, which is essential to achieve the crosslinking between target and effector cells. Therefore, CD20^+^ lymphoma cells were first incubated with the [CD20×NKG2D] bibodies, and then with soluble human NKG2D-Fc or the control protein NKp30-Fc. Cell-bound NKG2D-Fc fusion protein was subsequently detected with a secondary antibody conjugate directed towards the human Fc domain. Detection of the bibody/NKG2D-Fc complex was only possible when the [CD20×NKG2D] bibody bound both, cellular CD20 and soluble NKG2D-Fc simultaneously. The mean value of fluorescence intensity from experiments without adding bibody was calculated and clones demonstrating mean fluorescence values increased by at least a factor of 1.5 above that value were rated as binders. As indicated by shifts in mean fluorescence intensity, the different NKG2D-specific bibodies reacted with both CD20 and NKG2D, except one clone (**Figures 3A, B**). Interestingly, varying fluorescence intensity values were obtained with different clones, which may reflect different affinities to NKG2D. In contrast, after incubation with the control molecule NKp30-Fc no binding was detectable confirming the specificity of the [CD20×NKG2D] bibodies.

An important function of NK and T cell engagers is their capacity to activate the redirected immune effector cell population. Hence, the remaining 36 [CD20×NKG2D] bibodies were analyzed for their ability to activate human NK cells. Therefore, NK cells and lymphoma cells were incubated in the presence of the bibodies. Expression of the early activation marker CD69 on NK cells was only significantly induced by four [CD20×NKG2D] bibody constructs (**Figure 3C**). These data demonstrate that NKG2D engagement-induced activation is not a common feature of all NKG2D antibodies in the bibody format. In the following experiments we focused on bibodies containing anti-NKG2D scFv clones #3 (in the following figures indicated with blue color) and #32 (red color), which showed the highest NK cell activation efficiency. Clone #24 (green) was chosen as representative control for bibodies with low activation profile.

### 3.4 Cytotoxic capacity and synergistic activity in combination with non-engineered and Fc-engineered antibodies

**Figure 4:**
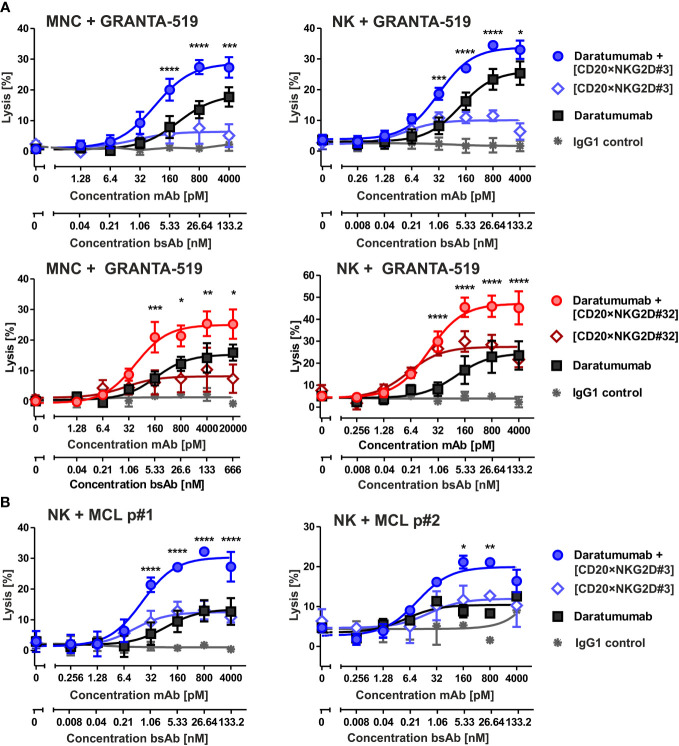
Cytotoxicity of the bibodies [CD20×NKG2D#3] and [CD20×NKG2D#32] and synergy with the CD38 specific mAb daratumumab. **(A)** CD20^+^/CD38^+^ GRANTA-519 MCL cells were incubated either with daratumumab, with the bispecific [CD20×NKG2D] antibodies or with their combinations, respectively, in presence of mononuclear cells (MNC; E:T ratio: 40:1) or NK cells (E:T ratio = 10:1) as effector population. After 4 h lysis of target cells was analyzed. The data points represent mean values of three independent experiments ± SEM. (*, statistically significant differences to treatment with daratumumab only; p ≤ 0.05). **(B)** [CD20×NKG2D#3] enhances ADCC triggered through daratumumab against tumor cells derived from two different MCL patients (p). NK cells were used as effector population. Data points represent the mean value from two independent experiments ± SEM (*, statistically significant differences to treatment with daratumumab only; p ≤ 0.05). **, P values between 0.001 and 0.01; ***, P values between 0.0001 and 0.001; ****, P values less than 0.0001.

**Figure 5:**
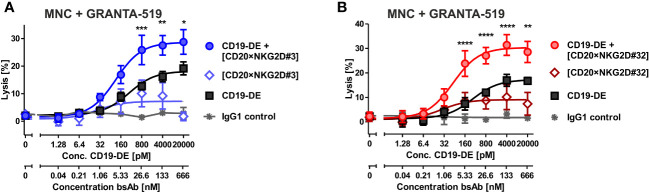
Cytotoxicity of combinations of bibody [CD20×NKG2D#3] and [CD20×NKG2D#32] with an Fc-engineered CD19 mAb (CD19-DE). The cytotoxic function of the bispecific antibodies [CD20×NKG2D#3] **(A)** and [CD20×NKG2D#32] **(B)** alone, or in combination with the Fc-engineered CD19-DE mAb, was analyzed in 4 h ^51^Cr release assays. GRANTA-519 MCL cells (CD19^+^, CD20^+^) were used as target cells and MNC isolated from healthy donors were applied as effector population (E:T ratio = 40:1). A non-binding monoclonal IgG1 Ab was used as a control. The data points represent the mean value of four independent experiments ± SEM. (*, statistically significant differences against the treatment with CD19-DE only; p≤ 0.05). **, P values between 0.001 and 0.01; ***, P values between 0.0001 and 0.001; ****, P values less than 0.0001.

In previous studies, we have shown that bispecific immunoligands engaging NKG2D trigger NK cell cytotoxicity and enhance NK cell-mediated ADCC by therapeutic antibodies (33, 34). To investigate whether the novel [CD20×NKG2D] bibodies exerted this function, the two bibodies with the highest activatory activity were either analyzed as single agents or were combined with the CD38 antibody daratumumab, and cytotoxicity was analyzed with both MNC or purified NK cells. CD38^+^/CD20^+^ mantle cell lymphoma (MCL) GRANTA-519 cells or lymphoma cells freshly isolated from two MCL patients were used as target cells. Both, bibody [CD20×NKG2D#3] and [CD20×NKG2D#32] used as single agents, induced lysis of GRANTA-519 MCL cells and primary lymphoma cells with MNC and purified NK cells as effector cell population, although to a moderate extent (**Figures 4A, B**). Importantly, the combination of the CD38 antibody and CD20-directed bibodies [CD20×NKG2D#3] or [CD20×NKG2D#32] was, with 33.0% and 45.2%, significantly more effective in triggering effector cell killing of tumor cells than the single agents. This was the case both when the cell line GRANTA-519 (**Figure 4A**) or isolated tumor cells from MCL-patients were analyzed (**Figure 5B**). Interestingly, the [CD20×NKG2D#24] bibody with low NK cell activation capacity in terms of CD69 induction was not able to increase tumor cell lysis in combination with monoclonal antibodies (**Supplementary Figure 2**).

Fc-engineering of mAbs by increasing their affinity to FcγRIIIA is a powerful method to augment their cytotoxic potential (7). To analyze whether the cytotoxic capacity of Fc-optimized antibodies could be enhanced by our novel agents, the Fc-engineered CD19 antibody (CD19-DE), which was modified for enhanced binding to activating FcγR (50), was tested in combination with the novel NK cell activating [CD20×NKG2D] bibodies in ADCC reactions. The co-stimulation of NKG2D with these bibodies enhanced CD19-DE-mediated ADCC against CD19^+^/CD20^+^ GRANTA-519 MCL target cells significantly and synergistically, even beyond the dose independent cytotoxicity plateau of single CD19-DE treatment (**Figure 5**, **Table 1**). Thus, in comparison to CD19-DE as single agent, the maximum lysis was increased from 18.2 ± 1,1% to 28.7 ± 1,9% by [CD20×NKG2D#3] and from 17.2 ± 1.7% to 30.3 ± 2,1% by [CD20×NKG2D#32], respectively. In conclusion, NKG2D co-stimulation synergistically enhanced mAb-mediated ADCC throughout all combination experiments (**Table 1**).

**Table 1:** CI values for combinations of monoclonal antibodies with the [CD20×NKG2D] bibodies.

Combination	Targets	Effectors	CI values at lysis of	
			5%	10%
Dara + [CD20×NKG2D#3]	GRANTA-519	MNC	0.31	n.a.
Dara + [CD20×NKG2D#3]	GRANTA-519	NK cells	0.68	0.17
Dara + [CD20×NKG2D#3]	MCL p#1	NK cells	0.59	0.24
Dara + [CD20×NKG2D#3]	MCL p#2	NK cells	2.47	0.22
Dara + [CD20×NKG2D#32]	GRANTA-519	MNC	0.42	n.a.
Dara + [CD20×NKG2D#32]	GRANTA-519	NK cells	0.49	1.52
CD19-DE + [CD20×NKG2D#3]	GRANTA-519	MNC	0.58	n.a.
CD19-DE + [CD20×NKG2D#32]	GRANTA-519	MNC	0.45	n.a.

Combination index (CI) values were calculated from dose response curves using the indicated target and effector cells for two different effect levels using GraphPad Prism 5.0 software. Not available values (n.a.), could not be calculated since not all single agents reached the required threshold (Dara, daratumumab).

### 3.5 Co-stimulation of bispecific T cell engagers

**Figure 6:**
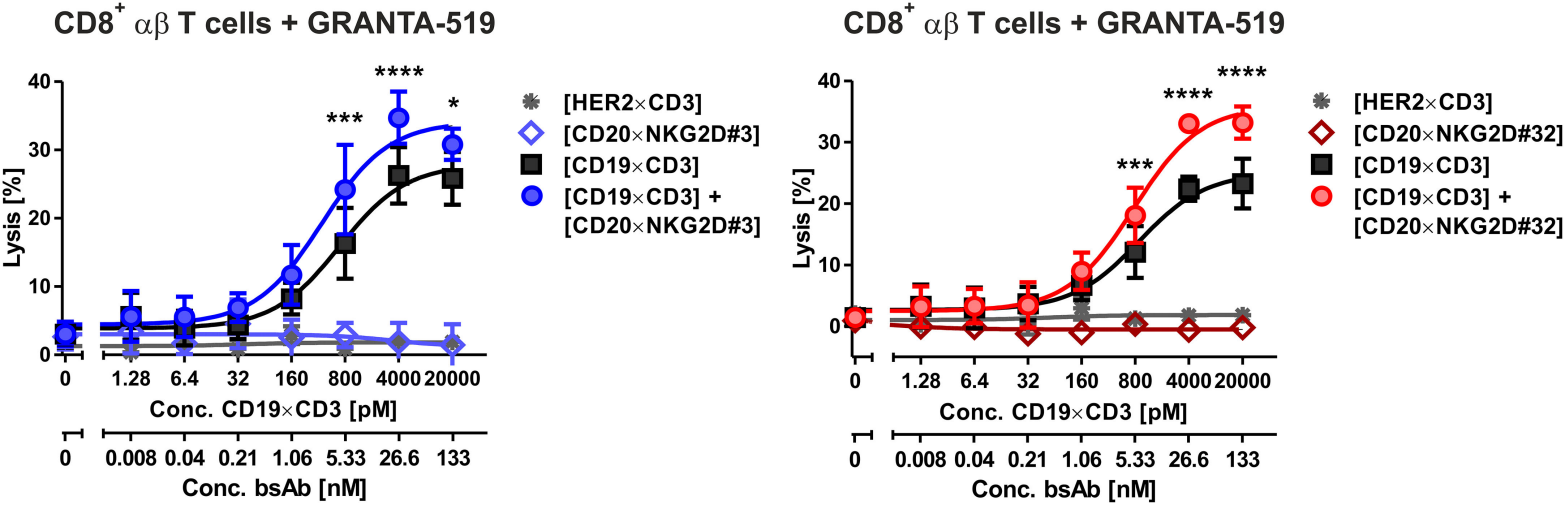
Cytotoxic activity of bispecific [CD20×NKG2D] antibodies with CD8-positive αβ T cells as effector population. CD8^+^ αβ T cells were isolated via MACS. The purity was determined by flow cytometry using CD3, CD8, CD16 and CD56 antibodies labelled with appropriate fluorescent dyes. Purified T cells were stimulated with IL-2 (300 U/ml) for three days and were tested as effector cells (E:T ratio: 20:1) for the bispecific antibodies [CD20×NKG2D#3] (left graph) and [CD20×NKG2D#32] (right graph) as well as their combinations with a [CD19×CD3] BiTE in a 4 h ^51^Cr release assay. GRANTA-519 MCL cells were used as target cells. The data points represent the mean value of three independent experiments ± SEM. (*, statistically significant differences against the treatment with [CD19×CD3] only; p ≤ 0.05). A [HER2×CD3] BiTE construct that does not bind to the target cells was used as a negative control. ***, P values between 0.0001 and 0.001; ****, P values less than 0.0001.

NKG2D is also expressed on CD8^+^ αβ T cells. We demonstrated in previous studies, that NKG2D-directed bispecific immunoligands were able to induce lysis of lymphoma cells by γδ T cell lines (51), but had low activity levels with αβ T cells (34). To investigate the potential T cell stimulatory function of the novel bibodies, T cell-mediated tumor cell killing triggered by bibodies [CD20×NKG2D#3] and [CD20×NKG2D#32] alone or in combination with a [CD19×CD3] bsAb was analyzed. Interestingly, both [CD20×NKG2D] bibodies significantly increased T cell-mediated lysis of GRANTA-519 MCL target cells from 26,3% ± 3,2 to 34,7 ± 3,3% and from 22,4 ± 3,0% to 33,0 ± 2,8% respectively, in combination with the [CD19×CD3] bsAb. As single agents the bibodies did not stimulate cytotoxicity of CD8^+^ αβ T cells (**Figure 6**).

In conclusion, bispecific antibodies, based on two novel anti-NKG2D human scFv antibodies were able to trigger NK cell cytotoxicity as single agents at moderate levels. Importantly, the bibodies were able to enhance ADCC of other tumor specific mAbs with non-engineered or engineered Fc as well as tumor cell killing by CD8^+^ cytotoxic T cells in combination with T cell engagers beyond the maximum level mediated by FcγRIIIA or CD3 triggering, respectively.

## 4 Discussion

In recent years, combination strategies of antibodies or their derivatives with either blockade of inhibitory immune checkpoint receptors or addition of a co-stimulatory signal through activation of a second trigger molecule on effector cells have gained particular interest in cancer immunotherapy. In this regard, co-stimulation of FcγRIIIA and NKG2D was reported to be an attractive strategy for the improvement of NK cell-mediated ADCC (33, 52). In this study, we isolated two novel NKG2D-specific bibodies and used them in combination with an approved non-engineered antibody. We further demonstrated synergistic action of these bibodies to enhance the cytotoxic capacity of Fc-engineered mAbs and a [CD19×CD3] T cell engager.

NKG2D and its ligands have received growing attention in immunotherapy of cancer (53). Current approaches include strategies to increase the expression of NKG2D ligands on tumor cells (54) or to trigger NKG2D signaling with antibody-derivatives. Moreover NKG2D ligands are investigated as target structures on tumor cells for different formats of therapeutic antibodies or CAR T cells (55–58). In this study, we identified 38 fully human NKG2D-specific scFv antibodies by phage display selection from the human antibody libraries HAL7 and 7b. These antibodies covered six different combinations of V_H_ and V_L_ families with high sequence diversity in their CDRs. The derived bibodies bound simultaneously to CD20 and NKG2D confirming their functional dual antigen binding. However, there were huge differences in the binding intensities measured by flow cytometry, which indicates different affinities of the scFv moieties to cell-presented NKG2D. These differences likely result from the NKG2D-specific scFvs, since all bibodies were constructed in the same format using the same CD20-specific Fab. Interestingly, the level of measured binding was not associated with the capacity to induce NK cell activation. The bibodies containing one of the two different NKG2D specific scFv antibody clones #3 and #32 induced high levels of NK cell activation, whereas NKG2D binding was only average. Previous studies demonstrated that affinity and avidity of bispecific NK cell engagers to their corresponding tumor antigen play a crucial role in terms of their efficacy (59, 60). Although speculative and not directly proven, our findings related to high NK cell activation by NKG2D scFvs with only average binding activity may be in line with a “hit rate” model of activation proposed for T cell engagers. Therefore, high affinity of the arm targeting the activating NKG2D receptor on NK cells may not enhance cytolytic activity above a certain maximum level and may even be detrimental as described for CD3-directed T cell engagers (61–63). Furthermore, the topology of the recognized NKG2D epitope and resulting orientation of the bispecific antibody may be different between the different clones which could also influence the bispecific cross-linking and NK cell activation. Nevertheless, bibodies based on clones #3 and #32 were able to induce NK cell-mediated tumor cell lysis by triggering NKG2D as single agents. Interestingly, [CD20×NKG2D#32] was more efficient in inducing NK cell-mediated tumor cell lysis than the bibody [CD20×NKG2D#3], despite its slightly lower activation levels.

Of note, we observed that both [CD20×NKG2D#3] and [CD20×NKG2D#32] bibodies enhanced NK cell-mediated ADCC of tumor specific mAbs - promoting the “dual-dual-targeting” concept, which we already proposed in the context of NKG2D-specific immunoligands (33). This approach based on re-targeting immune effector cells by linking two different activating or co-stimulatory receptors on effector cells with two different antigens on tumor cells may enhance tumor selectivity and cytotoxicity. We chose CD19 or CD38 as second tumor target, because both antigens are co-expressed with CD20 on a variety of B cell lymphomas or leukemias making these tumor antigen combinations ideal for the dual-dual-targeting approach. In our study, we showed that two novel [CD20×NKG2D] bibodies enhanced the ADCC of mAbs targeting CD19 or CD38 on the same tumor cells, thus, demonstrating the synergy of the NKG2D and FcγRIIIA mediated dual NK cell activation and the dual targeting of the CD20^+^/CD19^+^ or CD20^+^/CD38^+^ tumor cell lines.

Moreover, the bibodies [CD20×NKG2D#3] and [CD20×NKG2D#32] were able to even enhance the NK cell-mediated ADCC by the Fc-engineered antibody CD19-DE, which was optimized for FcγRIIIA binding and enhanced ADCC activity (50). This is an important finding, because in previous studies ADCC mediated by Fc engineered antibodies could not be enhanced above a certain threshold by further increasing the affinity of the engineered Fc to FcγRIIIA (64). However, here we could show, that simultaneous NKG2D activation may overcome this maximum level of cytotoxicity triggered by the FcγRIIIA receptor. Moreover, both bibodies were also able to enhance the cytotoxicity of re-targeted CD8^+^ T cells when applied in combination with a [CD19×CD3] BiTE (12, 13). Although the increase in cytotoxic activity was moderate further investigation of this combinatorial approach may be interesting because our actual test system may not reveal the whole spectrum of T cell effector functions triggered by the antibody combination. For example, analysis of cytokine release will be interesting since the level and profile of cytokine production may not directly correlate with cytotoxic activity, as recently shown by our group for NK cell engagers triggering two different receptors in the effector cell (65). Together, these findings suggest that the “dual-dual-targeting approach” may not only be applicable to monoclonal antibody therapy to enhance FcγRIIIA-mediated NK cell cytotoxicity but also to cytotoxic T cells when combined with CD3 targeting T cell engagers. Whether bispecific NKG2D antibodies will also be able to provide co-stimulatory signals to enhance TCR-mediated activation and thereby promote adaptive T cell responses as described for natural NKG2D ligands (27) remains to be determined.

Our data indicate that NKG2D and its different signaling cooperates synergistically with FcγRIIIA in NK cells and CD3 in T cells. As NKG2D contains an immunoreceptor tyrosine based activation motif (ITAM)-independent intracellular signal cascade by pairing with DAP10 (21), we assume that the activation of two distinct intracellular pathways leads to the synergistic effects between NKG2D and either FcγRIIIA or CD3, which both signal via ITAM motifs of the associated FcϵRIγ and/or CD3ζ polypeptides (66). This conclusion is supported by previous studies in which NKG2D enhanced FcγRIIIA mediated effects, whereas another NK cell receptor, natural killer protein 46 (NKp46), which signals through the same intracellular signal cascade as FcγRIIIA, did not (67). However, recently trifunctional NK cell engagers, which consisted of two antibody binding domains, one directed to a tumor associated antigen and one directed to NKp46, and the human Fc domain, showed enhanced cytotoxic properties, which suggests also a cooperation between NKp46 and FcγRIIIA and may be caused by additional factors, which remain to be uncovered (68).

Both T cells and NK cells are regulated by inhibitory receptors which may be employed as target structures for immune modulation. Thus, specific blockade of the immune checkpoints cytotoxic T-lymphocyte-associated protein 4 (CTLA-4) or programmed cell death protein 1 (PD-1) has shown potential in the treatment of different types of cancer by promoting T cell responses in patients. Also in NK cells a number of candidate inhibitory receptors have been identified as potential targets for immune checkpoint blockade (69). Besides human killer cell immunoglobulin-like receptors (KIR), T cell-activated increased late expression (TACTILE), T cell immunoreceptor with Ig and ITIM domains (TIGIT) and others, in particular natural killer group 2 member A (NKG2A) may represent an interesting target, since its blockade enhanced NK cell-mediated ADCC (70, 71). However, the impact of these inhibitory receptors on the proposed “dual-dual-targeting” approach relying on concomitant activation of FcγRIIIA and NKG2D in NK cells or CD3 and NKG2D in T cells has not been analyzed yet. It would be interesting to assess whether cytotoxicity may be enhanced even further by simultaneous co-blockade of such inhibitory receptors.

In conclusion, screening of human antibody libraries led to the identification of a panel of NKG2D-specific scFv antibodies. Two scFvs showed favorable characteristics when used as NK and T cell activating or co-stimulatory moieties in dual specific [CD20×NKG2D] bibodies. The novel bibodies enhanced cytotoxicity of NK and T cells, particularly in combination with mAbs or bispecific T cell engagers targeting the same tumor and effector cell type. This novel dual-dual targeting approach of two different cytotoxic signal mechanisms in NK and T cells in combination with targeting two different tumor antigens led to a higher maximum level of cytotoxicity. This maximum was not achieved with higher doses of the single agent. Bispecific antibodies as described in this study can potentially offer a broad application to potentiate immunotherapies employing mAbs or NK/T cell engagers which trigger cytoxicity via FcγRIIIA or CD3, but especially studies in preclinical animal models are required to explore their full potential.

## Data availability statement

The raw data supporting the conclusions of this article will be made available by the authors, without undue reservation.

## Ethics statement

The studies involving humans were approved by Ethics committee of the medical faculty of the Christian Albrechts Univerity of Kiel; Haus U 27 Schwanenweg 20; D - 24105 Kiel. The studies were conducted in accordance with the local legislation and institutional requirements. The participants provided their written informed consent to participate in this study.

## Author contributions

SL, A-MA, LE, HT, AL, DW, SK, KK, RS designed and performed the experiments and/or analyzed data and discussed the data. performed the research and contributed to writing the manuscript. AF, MH, TS, SD generated and provided essential reagents, contributed to the design of the study and contributed to writing the manuscript. CK, SL, MP, MG, TS outlined the research project. All authors contributed to writing, editing and discussing the manuscript. All authors contributed to the article and approved the submitted version.
