# Supplementary material for: Novel NKG2D-directed bispecific antibodies enhance antibody-mediated killing of malignant B cells by NK cells and T cells
Source: Front Immunol. 2023 Oct 27;14:1227572. doi: 10.3389/fimmu.2023.1227572 (PMC10641740; doi:10.3389/fimmu.2023.1227572)
Supplement: Supplementary file 1 [file DataSheet_1.docx]

**Supplementary Material**


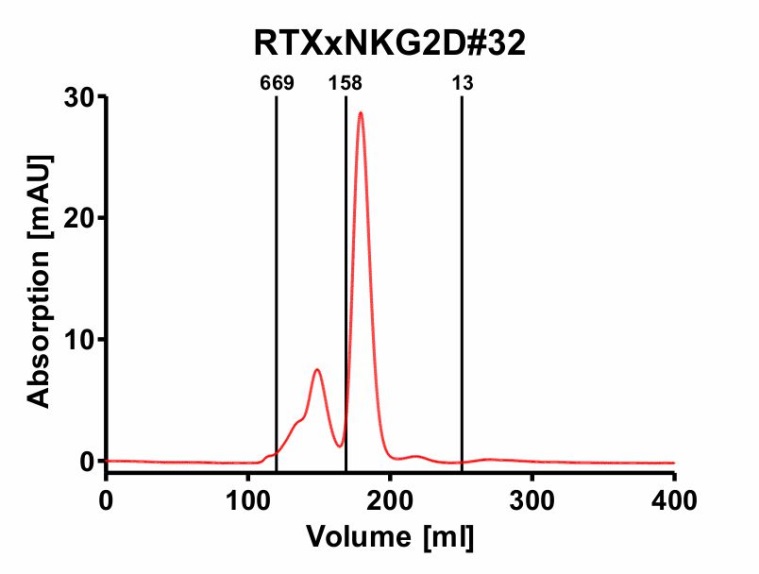


**Supplementary Figure 1. Size exclusion chromatography (SEC) of the bibody [CD20×NKG2D#32].** The bibody preparation was analyzed by SEC and compared to a molecular weight standard (669 kDa, 158 kDa, 13 kDa). One representative experiment is shown.


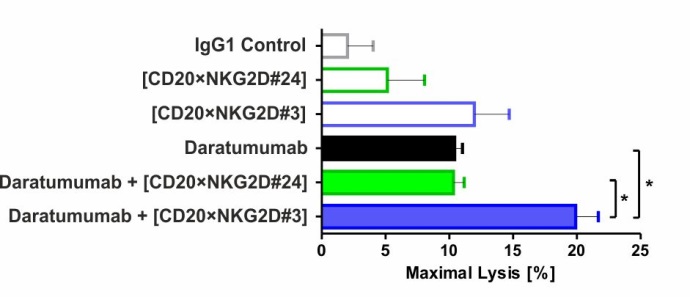


**Supplementary Figure 2. The bibody [CD20×NKG2D#24] based on a non-activating NKG2D scFv does not trigger cytotoxicity or co-stimulatory function.** The cytotoxic function of the bibody [CD20×NKG2D#3] and [CD20×NKG2D#24] alone or in combination with daratumumab, was analyzed in a 4 h ^51^Cr release assay. GRANTA-519 MCL cells (CD38^+^, CD20^+^) were used as target cells and MNC isolated from healthy donors as effector population (E:T ratio: 10:1). A non-binding monoclonal IgG1 Ab was used as a control. The data points represent the mean value of three independent experiments ± SEM. (*, statistically significant differences are indicated; p≤ 0.05).


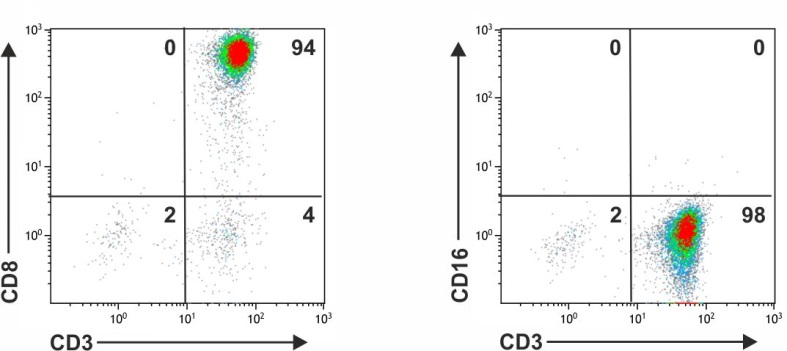


**Supplementary Figure 3. Purity of CD8^+^ T cell preparations.** CD8^+^ T cells were purified from the MNC fraction by negative selection using the MACS technology and stained with specific antibodies against CD8 and CD3 (left dot plot). The presence of NK cells in T cell preparations was excluded by staining against CD16 (FcγRIII) and CD3 (right dot plot). One representative experiment is shown.
